# Anti-Adhesive Organosilane Coating Comprising Visibility on Demand

**DOI:** 10.3390/polym14194006

**Published:** 2022-09-24

**Authors:** Wolfgang Kern, Matthias Müller, Christine Bandl, Nina Krempl, Markus Kratzer

**Affiliations:** 1 Montanuniversität Leoben, Chair in Chemistry of Polymeric Materials, Otto-Glöckel-Straße 2, A-8700 Leoben, Austria; 2 Montanuniversität Leoben, Chair in Polymer Processing, Otto-Glöckel-Straße 2, A-8700 Leoben, Austria; 3 Montanuniversität Leoben, Chair in Physics, Franz-Josef-Straße 18, 8700 Leoben, Austria

**Keywords:** anti-adhesive, coating, organosilane, fluorescence, visibility on demand, naphthalimide

## Abstract

There is a wide application field for anti-adhesive and hydrophobic coatings, stretching from self-cleaning surfaces over anti-graffiti and release coatings to demolding aids in the production of polymers. The typical materials for the latter are hard coatings, including TiN, CrN, diamond-like carbon, etc. Alternatively, organosilane coatings based on perfluorinated compounds or molecules with long alkyl side chains can be employed. Although these functional layers are generally required to be invisible, there is a demand for a straightforward approach, which enables the temporary control of successful and homogeneous application as well as abrasion and wear of the coatings during use. For this purpose, a visibility-on-demand property was introduced to an already established anti-adhesive organosilane coating by incorporation of 1,8-naphthalimide-N-propyltriethoxysilane (NIPTES) as a fluorescent marker molecule. While the naphthalimide unit provides blue fluorescence under UV irradiation, the ethoxy groups of NIPTES enable the covalent coupling to the coating as a result of the hydrolysis and condensation reactions. As a consequence, the fluorescent marker molecule NIPTES can simply be added to the coating solution as an additional organosilane component, without the need for changes in the approved deposition procedure. The generated fluorescent anti-adhesive coatings were characterized by contact angle measurements, atomic force microscopy (AFM), as well as by different spectroscopic techniques, including FTIR, UV-Vis, fluorescence and X-ray photoelectron spectroscopy (XPS). In addition, the on-demand control function provided by the introduced fluorescence properties was evaluated along an injection molding process.

## 1. Introduction

The wide application field for anti-adhesive and hydrophobic coatings covers self-cleaning surfaces [1,2], anti-graffiti [3,4] and release coatings for adhesive tapes and labels [5,6], as well as demolding aids in the production of polymers [7,8]. These coatings rely either on microstructural effects (e.g., lotus effect) [2] or chemical surface modification. Concerning the latter, fluorinated organic compounds in particular proved to be suitable for the introduction of hydrophobic and anti-adhesive surface properties [9,10,11,12]. This can be derived from the high difference between the electronegativity of carbon (2.5) and fluorine (4.0), which results in high bond strength (binding energy = 485.7 kJ/mol) and low polarizability of the C–F bond [13,14,15].

**Figure 1:**
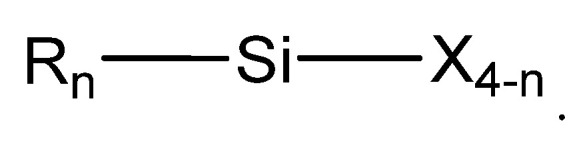
General structure of organosilanes.

Since their bifunctional structure (see Figure 1) enables interactions and chemical reactions with both organic and inorganic materials [16], organosilanes are well known as surface modifiers, coupling agents [16,17] and protective layers [18,19]. Regarding surface modification, the hydrolyzable groups X are employed for coupling to inorganic surfaces, while the organofunctional groups R determine the properties of the modified substrate. During coating, the organosilane molecules undergo a series of hydrolysis and condensation reactions [20]. Initially, the moieties X are hydrolyzed stepwise in the presence of water, building the corresponding silanols, which subsequently adsorb to superficial OH-groups of the substrate via hydrogen bonding. In the subsequent condensation reaction, the silanols are covalently bound to the substrate surface under the elimination of water, resulting in siloxane bonds. Simultaneously, there is a competitive reaction with adjacent silanols, which generates a polysiloxane network at the surface [16,17,21,22,23]. Typical hydrolyzable groups are halogen moieties as well as alkoxy units, including methoxy and ethoxy groups [16,17]. Examples of hydrophobic organofunctional groups are long alkyl chains and fluoroalkyl moieties [9,13,19,20,24,25].

In general, coatings and damages of the same can be detected by various characterization methods, such as (i) microscopy (scanning electron microscopy, atomic force microscopy, imaging confocal microscopy, etc.), (ii) spectroscopy (X-ray photoelectron spectroscopy, Fourier transform infrared spectroscopy) and (iii) interferometry (phase shifting interferometry and coherence scanning interferometry) [26]. Moreover, contact stylus profilometry, contact angle measurements, thermal imaging, electrochemical measurements, ultrasonic inspection, acoustic emission and radiography can be employed. However, these techniques are time consuming, costly, require bulky equipment and an external energy supply, and are often very complex, which makes them impractical for detection over large areas and also unsuitable for application in industrial processes. Alternatively, fluorescent, color-changing or mechanically triggered indicators can be incorporated into coatings [27].

**Figure 2:**
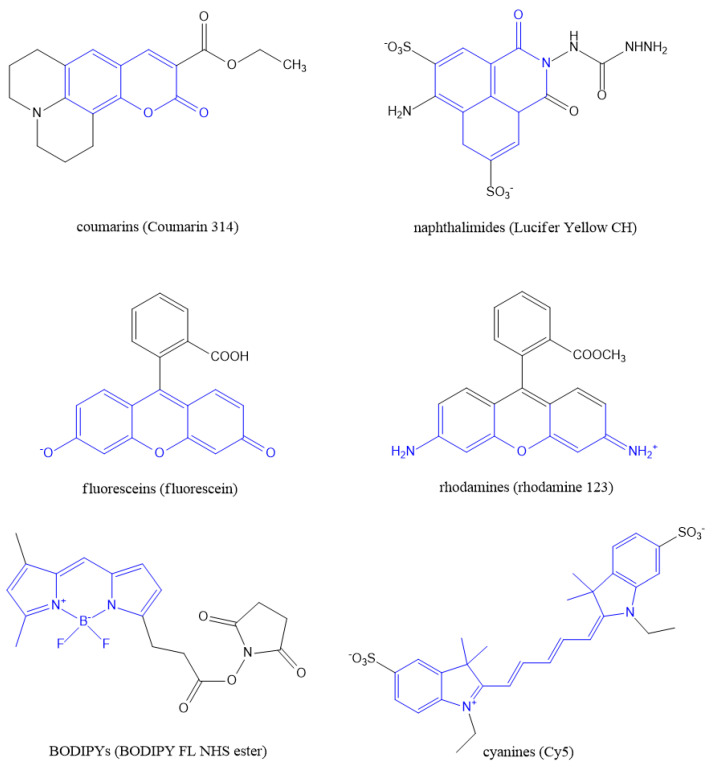
Representative examples of fluorophores used for labeling in biology and medicine (adopted from Ref [31]).

Fluorescent markers and labels are commonly used for localization of biomolecules and to examine biological processes, such as protein interactions, enzymatic activity, conformation changes and real-time movement of proteins [28,29,30]. In addition to these cellular and peptide studies, fluorescent markers are also used for the discovery of new drugs, environmental analysis and medical applications, including the detection of cancer [31]. Upon absorption of a photon, the marker molecule is excited from the ground singlet state S_0_ to an excited state (S_1_ or S_n_). Then, it relaxes to the lowest vibrational level of the first excited state S_1_ (via non-radiative processes) and finally emits fluorescent light when relaxing to the ground state S_0_ [28,30,31]. Important representatives of fluorophores commonly used for labeling include coumarins, naphthalimides, fluorescein and its derivatives, rhodamine and its analogs, BODIPY dyes (boron difluoride unit attached to the dipyrromethane group) and cyanines (see Figure 2) [31,32,33]. Moreover, the green fluorescent protein (GFP) [29,34] and its derivatives, including the blue, cyan, red and yellow fluorescent protein [33,35,36], are employed for labeling. Alternatively, the nanoparticles and quantum dots of inorganic semiconductor nanocrystals can be used for fluorescent imaging [28,29,30]. The suitable nanoparticles consist of noble metals (mainly Au and Ag), fluorescently doped silica, up-conversion materials, such as NaYF_4_ or KMnF_3_ doped with lanthanides Er(III), Yb(III) or Tm(III), or metal chalcogenides, such as CdSe [32,33,37].

In addition to the above-described labeling molecules, organosilanes comprising fluorescent moieties are also employed as optical markers. For example, Ref [38] describes the use of fluorescent organosilanes to control the uniformity of a primary silane or thiol-based layer, which is applied to immobilize biomolecules on a microarray. Beyond the field of biology and medicine, fluorescent organosilane compounds are deployed for the detection of explosives and pesticides, where NOx-containing analytes are identified by interactions with fluorescent silanes bearing arylamine groups [39]. Moreover, an epoxy-functional silane was labeled with a dimethylaminonitrostilbene fluorescent dye and added to the coupling agent layer in order to study the resulting polymer/coupling agent/substrate interface [40,41]. Another example encompasses the photo-chemically induced modification of nanocrystalline diamond films with N^1^-(3-(trimethoxysilyl)propyl)hexane-1,6-diamine, which were proposed for biosensor applications [42]. As an alternative to the addition of fluorescent markers, color-changing indicators (e.g., common pH indicators, such as phenolphthalein, or metal ion indicators, including phenanthroline) or mechanochromic indicators (e.g., spiropyranes) can also be incorporated as described for self-reporting corrosion protection coatings in Ref [27]. Another approach relies on the inherent fluorescence of the selected polymer matrices. One example is represented by a corrosion protection coating based on a poly(phenylene methylene) (PPM) copolymer. The UV-stimulated fluorescence of the phenylene units enables the optical detection of inhomogeneities, cracks and other defects, which are caused by pit attacks [43].

In this work, the concept of fluorescence labeling is transferred to the field of low surface energy coatings by extending an already established anti-adhesive layer by a visibility-on-demand property. Therefore, the silane-based marker 1,8-naphthalimide-N-propyltriethoxysilane (NIPTES) bearing a naphthalimide moiety as well as a triethoxysilyl group was synthesized and deposited together with fluoroalkyl organosilanes of the anti-adhesive coating. While the naphthalimide unit in NIPTES provides blue fluorescence under UV irradiation, the ethoxy groups of NIPTES enable covalent coupling to the coating resulting from hydrolysis and condensation reactions. This represents a quick and straightforward approach to temporarily monitor the presence and homogeneity of the applied coating as well as its damage during use. For the general characterization of the organosilane coatings by means of XPS, FTIR, UV-Vis and fluorescence spectroscopy, Si wafers were chosen as substrates. The silicon surface is representative of the different inorganic surfaces, such as metals, ceramics and oxides, to which anti-adhesive coatings in their function as demolding aids, self-cleaning as well as anti-graffiti, anti-fogging and anti-icing coatings can be applied. In addition, steel substrates were coated to demonstrate the anti-adhesive property of the coatings and the on-demand fluorescence control function, which were evidenced by adhesion force measurements (with AFM) and along an injection molding process, respectively.

## 2. Materials and Methods

### 2.1. Materials

The organosilanes 1,8-bis(triethoxysilyl)octane (95%; BOS) and 1H,1H,2H,2H-perfluorooctyltriethoxysilane (97%; PFOS) were purchased from abcr GmbH (Germany) and used without further purification. Ethanol (absolute, 99.5%; EtOH) was acquired from VWR International (United States), and acetic acid (rotipuran, 100 %; AcOH) was obtained from Carl Roth GmbH + Co. KG (Germany).

For the synthesis of 1,8-naphthalimid-N-propyltriethoxysilane (NIPTES), 1,8-naphthalic anhydride and 3-aminopropyltriethoxysilane (99%) were purchased from Sigma-Aldrich GmbH (Germany) and used without further purification. Moreover, anhydrous ethanol (99,8%) from VWR International (United States) was used as a solvent.

Single-side finished silicon wafers (with one side being polished and reflecting) were kindly provided by Infineon Technologies Austria AG (Villach, Austria).

Stainless steel (DIN 1.2343; X38CrMoV5-1) substrates (66 mm × 80 mm) were kindly provided by Poloplast GmbH & Co KG (Austria). Typically, this steel type contains 0.38 wt% carbon, 1.1 wt% silicon, 0.4 wt% manganese, 5.0 wt% chromium, 1.3 wt% molybdenum and 0.4 wt% vanadium. The surface roughness was determined by AFM measurements (see Chapter 3.3).

A blue-colored chalk-filled polypropylene compound (PKNG-Spritzcompound) was also kindly provided by Poloplast GmbH & Co KG (Austria) and used as the injection molding material.

### 2.2. Synthesis of the Fluorescent Marker Molecule

**Figure 3:**
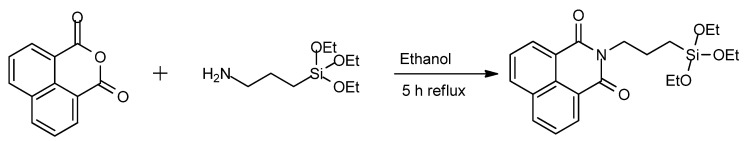
Synthesis of the fluorescent silane 1,8-naphthalen-imid-N-propyltriethoxysilan (NIPTES) from 1,8-naphthalic anhydride and 3-aminopropyltriethoxysilane.

As described in Ref [44], NIPTES was synthesized from 1,8-naphthalic anhydride and 3-aminopropyltriethoxysilane (APTES) in anhydrous ethanol. The corresponding condensation reaction is shown in Figure 3. An amount of 4 mmol 1,8-naphthalic anhydride was dissolved in 60 mL ethanol before 4 mmol APTES was added. The reaction solution was heated to 80 °C and was kept for 5 h under reflux, inert gas atmosphere (N_2_) and mechanical stirring. Subsequently, a brown gel was isolated by removing the solvent under vacuum. Finally, storage at room temperature under inert gas atmosphere led to crystallization of the synthesized NIPTES (brown solid). The product was characterized by NMR, FTIR and UV-Vis spectroscopy. FTIR and ^1^H NMR data were in accordance with the literature data [44].

^1^H NMR (CD_3_CD_2_OD, ppm): 0,7 (t, 2H, Si–CH_2_), 1,2 (t, 9H, CH_3_), 1,8 (q, 2H, CH_2_), 3,8 (quart, 6H, O–CH_2_), 4,14 (t, 2H, N(imid)–CH_2_), 7,8 (t, 2H, Ar), 8,3 (d, 2H, Ar), 8,5 (d, 2H, Ar)

FTIR spectroscopy (wave number, cm^−1^): 2975–2885 (-CH_2_-, -CH_3_); 1706, 1660 (-C=O), 1072 (-Si-O)

### 2.3. Sample Preparation

#### 2.3.1. Coating of Si Wafers

The Si wafers were immersed in isopropanol and acetone and ultrasonicated for 10 min. Then, the Si wafers were activated by an oxidizing flame treatment of the polished side (for details, see Ref [10]) and coated with different silane solutions. The coating solutions were prepared by dissolving PFOS, BOS and NIPTES in an EtOH/H_2_O mixture (9:1) at defined concentrations (compare Table 1). In addition, the solutions were acidified with AcOH (20 µL/10 mL) and ultrasonicated for 10 s. Then, the prepared solutions were stirred at 70 °C for 24 h or 48 h in order to provide a high degree of hydrolysis and a certain degree of condensation of the silanes. Next, the silane solution was drop cast onto the cleaned and activated Si wafers. The solvent was evaporated either at room temperature (20 °C), 70 °C or 180 °C before curing the silane coating at 180 °C for a period of 24 h. Finally, excess silane was removed by ultrasonication and wiping with acetone. These samples are termed coat_1_xx and coat_2_xx hereinafter, where 1 and 2 refer to the different compositions listed in Table 1, and xx represents the respective temperature for solvent evaporation (in centigrades). For comparison, reference samples without NIPTES were prepared from PFOS and BOS, as described above (termed ref_xx). In general, the coatings containing NIPTES appeared more homogeneous than the reference layers.

It has to be mentioned that although the prehydrolysis time of coat_1 differs from those of the reference and coat_2, comparisons between all silane layers are considered acceptable, since previous work has indicated that the prehydrolysis time only plays a minor role in coating formation.

#### 2.3.2. Coating of Steel Substrates for Injection Molding Experiments

Stainless steel plates, which were specially manufactured removable inserts for an injection molding machine, were cleaned by immersion in peroxymonosulfuric acid (Caro’s acid; prepared from H_2_O_2_ (30%) and concentrated H_2_SO_4_ at a ratio of 1:3) for 3 h to remove any organic residues of previous coating experiments. After washing with deionized water and drying, the steel substrates were activated by an oxidizing flame treatment on the polished side. The coating solutions for ref_180, coat_1_180 and coat_2_180 were prepared as described for the coating of Si wafers. However, for coating of the steel plates, about 50 % of the solvent was evaporated, and the concentrated organosilane solutions were applied via brush coating. Subsequently, the coatings were cured at 180 °C for 24 h. The variation of the application technique (brush coating instead of drop coating) was due to easier handling. Since the steel plates were much larger in size than the Si wafers, they were more difficult to handle during coating, which led to inhomogeneous organosilane layers.

### 2.4. Characterization Methods

#### 2.4.1. FTIR Spectroscopy

The synthesized fluorescent organosilane as well as the coated substrates were characterized by means of FTIR spectroscopy, using a Vertex 70 IR spectrometer and the corresponding software Opus Version 7, both from Bruker Optics (Germany). The spectra of neat NIPTES were recorded via the ATR unit, while the coated Si wafers were analyzed in transmission mode. All spectra were recorded, taking 16 scans at a resolution of 4 cm^−1^.

#### 2.4.2. NMR Spectroscopy

The synthesized NIPTES was also investigated by NMR spectroscopy, employing a Bruker Avance III spectrometer (700 MHz, Bruker Optics, Germany). All spectra were recorded at a temperature of 25 °C, and TMS was used as an internal standard. The evaluation of the NMR spectra was conducted using the MestReNova x64 software.

#### 2.4.3. UV-Vis Spectroscopy

In addition, NIPTES was characterized with regard to its absorption properties using a Cary 50 UV-Vis spectrometer (Varian, Australia), which was equipped with a xenon-flashlight lamp. The measurements were controlled via the corresponding Cary WinUV software. For this investigation, the solutions of NIPTES in ethanol at concentrations of 25–110 µmol/L were prepared and examined in the spectral range of 190–1100 nm.

#### 2.4.4. Contact Angle Measurements

Furthermore, the coated Si wafers were investigated by measuring the water contact angles (WCA) at room temperature, employing a drop shape analyzer (DSA 100, Krüss GmbH, Germany). Two-microliter droplets of ultrapure water were deposited on the sample surface. The WCA was averaged over three to five points per sample. Alternatively, a mobile surface analyzer (MSA, Krüss GmbH, Germany) was applied to record the changes of the WCA during application of the coated steel substrates in injection molding.

In order to ensure comparability, it was verified that the results of the mobile contact angle measuring device correlated well with the results of the drop shape analyzer.

#### 2.4.5. Fluorescence Spectroscopy

The emission properties of the deposited coatings were investigated by means of fluorescence spectroscopy using a Cary Eclipse spectrometer and the Cary Eclipse Scan Application software, both from Varian (USA). The samples were excited by UV irradiation at a wavelength of 365 nm, and the spectra were recorded at a scan rate of 600 nm per min. Since some samples showed too high fluorescence intensity, the intensity of the excitation light was adjusted.

#### 2.4.6. X-ray Photoelectron Spectroscopy (XPS)

The chemical composition of the sample surfaces was analyzed by XPS at room temperature using a Kα Thermo Scientific Photoelectron Spectrometer with monochromatic Al Kα radiation (1486.6 eV). The survey scan was carried out at a pass energy of 200 eV and an energy resolution of 1.0 eV, while the high-resolution spectra were recorded at a pass energy of 50 eV and a resolution of 0.1 eV. The C 1 s line was used to calibrate the binding energy scale for the measurements, assuming a binding energy of 284.8 eV for C–C bonds. Hydrogen was omitted in the calculation of the surface composition. For each sample, at least two measurements were performed. The data in Table 2 are averaged values.

#### 2.4.7. Spectroscopic Ellipsometry and Profilometry

Ellipsometric measurements were performed to determine the thickness of the organosilane layers deposited by drop coating. Therefore, an ex situ variable angle spectroscopy ellipsometer (VASE M-200, J.A. Woollam, Australia) was used. The thickness of the brush-coated organosilane layers was estimated by measuring the depth of manually prepared scratches in the coatings using a Dektak 150 profilometer (Veeco, USA), employing a tip with a radius of 12.5 µm.

#### 2.4.8. Atomic Force Microscopy (AFM)

The morphology of uncoated and coated Si wafers and steel substrates was examined utilizing an Asylum Research MFP 3D atomic force microscope (AFM) (Asylum Research, USA.) The system was equipped with an 85 × 85 μm^2^ x-y – scanner and a closed-loop 15 μm z-scanner. The topography measurements were performed in intermittent contact mode using Olympus AC160 TS AFM probes with a typical spring constant of 29 N/m and typical probe tip radii <7 nm. The RMS roughness (Ra) was calculated from the topography images using the scanning probe analysis free software Gwyddion [45].

Moreover, local adhesion force measurements were performed on the uncoated and coated steel substrates using the same AFM system. The spring constant was calibrated using the thermal sweep method (typically ∼25 N/m) [46]. A maximum contact force of ∼23.8 nN was applied with an approach and retract velocity of 1.5 μm/s. The adhesion force was determined by evaluating the force distance curves performed on a 64 × 64 point array over a 2 × 2 μm^2^ surface area [47]. The adhesion force values were automatically determined by the Asylum Research AFM software after calibration. At least three different positions were measured on each sample for better data statistics. In order to ensure tip stability throughout the measurements, bare steel samples were used as references.

#### 2.4.9. Injection Molding Experiments

The on-demand control function of the fluorescent organosilane coatings was demonstrated along an injection molding process. These measurements were performed by employing a special two-plate injection mold, which was developed within a joint project between PCCL and the Montanuniversität Leoben (Chair in Polymer Processing). Within this mold, the polymer melt is injected under pressure into the mold cavity in which a plate-shaped polymer part is produced. More details about the functional principle of this special injection mold can be found in Refs [48] and [49].

In this work, the polymer parts of a blue chalk-filled polypropylene compound were manufactured applying the following processing conditions: 240 °C nozzle temperature, 550 bar specific injection pressure, v/p switch over 99%, 180 bar packing pressure, 28 s packing time, 5 s residual cooling time. During the entire packing and rest cooling phase, as well as during the first 30 mm of the demolding stroke, a hydraulically driven vertical piston exerts a pressure of 44 bar onto the polymer part.

The injection molding experiments were carried out on steel plates coated with ref_180 (without NIPTES), coat_1_180 and coat_2_180 (see also Chapter 2.3). The mold surface temperature was adjusted to 30 °C, as recommended by the supplier of the polypropylene compound (according to the internal datasheet provided by Poloplast). The degradation of the coatings by abrasion and wear was followed by irradiation with a UV lamp after every 10th production cycle. Furthermore, the contact angle with water was measured at the same interval for all steel plates (uncoated, ref_180, coat_1_180 and coat_2_180).

## 3. Results

### 3.1. Deposition of Fluorescent Anti-Adhesive Coatings

**Figure 4:**
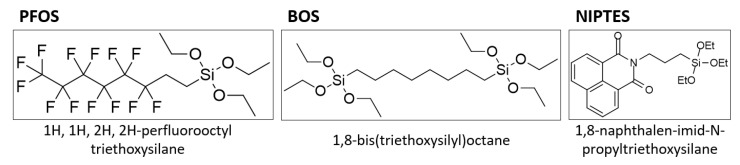
Chemical structures of the applied organosilanes PFOS, BOS and NIPTES.

As described in our previous studies, organosilane coatings based on PFOS and BOS comprising low surface energy were deposited onto metallic substrates by liquid phase deposition [10,48,50]. In the present work, the already established anti-adhesive coating based on PFOS and BOS (see Figure 4) was amplified by a visibility-on-demand property in order to introduce a quick and straightforward approach to temporarily monitor the presence and homogeneity of the applied organosilane layer.

For this purpose, the synthesized organosilane NIPTES (see Figure 4) was added to the coating solution and deposited together with PFOS and BOS on activated (i.e., oxidized) silicon wafers and steel substrates. While the naphthalimide moiety of NIPTES provides the required visibility-on-demand property (discussed in more detail in Chapter 3.3), the ethoxy groups enable the fluorescent marker to participate in hydrolysis and condensation reactions and, consequently, to become part of the siloxane network, which is covalently coupled to the substrate.

As can be derived from the molar ratios in Table 1, the samples of the series coat_1 contained 73.6 mole-% of NIPTES, while the samples of the series coat_2 contained 30.8 mole-%. In addition, a reference series without NIPTES was prepared. The significant variations of the PFOS/NIPTES ratio in the reference coating, in coat_1 and coat_2 enabled the investigation of the impact of the fluorescent marker on the optical and surface properties of the anti-adhesive coating. Moreover, the effect of the reaction temperature on the chemical composition was investigated by varying the evaporation temperature from 20 over 70 to 180 °C. Despite the different prehydrolysis times of coat_1, the comparisons described here were considered acceptable, since our previous work indicated that the prehydrolysis time only plays a minor role in coating formation. According to the ellipsometric and profilometric measurements, the thickness of the generated layers (drop cast and brush coated) was in the range of 1.5–2.5 µm.

### 3.2. Characterization of the Coatings by FTIR Spectroscopy and XPS Analysis

**Figure 5:**
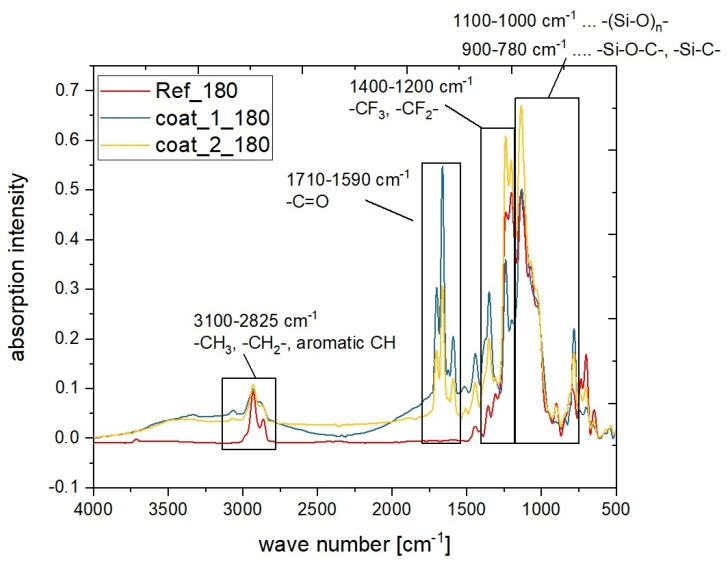
FTIR spectra of a reference coating (red) composed of PFOS and BOS (Ref_180), and representative coatings of coat_1 and coat_2 series composed of PFOS, BOS and NIPTES (coat_1_180—blue, coat_2_180—yellow).

The successful incorporation of NIPTES into the organosilane coating was proven by FTIR and XPS spectroscopy. Therefore, representative silane coatings containing NIPTES were compared to a reference layer based on PFOS and BOS. FTIR spectroscopy revealed typical peaks for the siloxane network (1100–780 cm^−1^) [9,44,51] and for fluorinated carbon chains CF_x_ (1400–1200 cm^−1^) [9,51,52] originating from PFOS in both the reference as well as in the NIPTES-containing coatings (see Figure 5). The incorporation of the fluorescent marker into the organosilane coating became evident from the FTIR bands in the range of 1710–1590 cm^−1^, which were only present in the spectrum of the coatings containing NIPTES. These FTIR bands are characteristic for the C=O units of imides [44,52]. Moreover, hydrocarbon units were detected in both the reference and the coatings containing NIPTES. In the reference coating, the signals at 3100–2825 cm^−1^ were caused by the alkylene units –(CH_2_)_n_ of PFOS and BOS only, while for the coatings containing NIPTES, the aromatic ring system of the naphthalimide moiety and the CH_2_ groups also contributed to these signals [52]. Furthermore, a comparison of the NIPTES-containing samples clearly displayed the differences in the chemical compositions of coat_1_180 and coat_2_180 (see Table 1). The higher ratio of NIPTES in coat_1_180 caused stronger imide peaks (1710–1590 cm^−1^) than those observed for coat_2_180. On the contrary, coat_2_180 showed more intense bands in the range of 1400–1200 cm^−1^ (CF_x_) originating from the higher amount of PFOS in this formulation.

**Figure 6:**
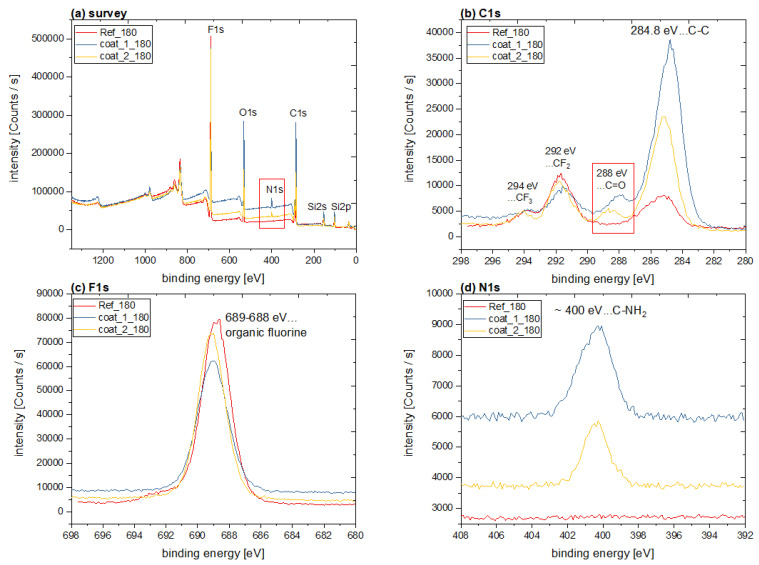
(**a**) XPS survey and (**b**–**d**) high-resolution spectra (C1s, F1s, N1s) of the reference and representative NIPTES-containing coatings.

The findings of FTIR spectroscopy were confirmed by the results of XPS measurements. The qualitative evaluation of the survey spectra (see Figure 6a) proved the deposition of the reference coating (PFOS + BOS) as well as the NIPTES-containing silane layers by the presence of Si, C, O and F. While the F signal is characteristic for the perfluorinated chains of PFOS, Si, C and O represent the siloxane network formed by all the applied organosilanes. In addition, N was detected for the NIPTES-containing samples exclusively, originating from the imide moiety of the fluorescent marker.

More detailed information about the chemical composition of the investigated surfaces was obtained from high-resolution spectra of C1s, F1s and N1s. In addition to the typical peak for organic silane compounds at 284.8 eV (Si-C, C-C), all the C1s spectra (Figure 6b) were characterized by the peaks at 292 eV and 294 eV, which are indicative of fluoroalkyl groups originating from PFOS. This was also reflected by the peak at 689 eV in the F1s spectra (Figure 6c), which is characteristic for organic fluorine compounds [9,10,53]. The incorporation of NIPTES was proven by the peaks at 288 eV and 400 eV in the C1s and the N1s spectra (Figure 6b,d), respectively. These signals were exclusively detected for the NIPTES-containing coatings and are characteristic for the C=O and C–N bonds of the imide moiety of the fluorescent marker [53,54,55,56]. Concerning the N1s spectra (Figure 6d), it has to be mentioned that the different intensities of the presented samples originate from the automated approach in the XPS measurement, whereby the sample surface is automatically positioned in the optimum height relative to the electron gun and the detector.

The results discussed above are further corroborated by the quantitative evaluation of the survey spectra (see Table 2), which were also compared to the theoretical composition of the coatings ref, coat_1 and coat_2. As already described, N was only detected for the NIPTES-containing coatings originating from the naphthalimide moiety of the fluorescent marker. This is also reflected quantitatively by the absence of N in the reference layers, while the surfaces of coat_1 and coat_2 samples contained 1.5–2.4 at% N. Further comparison of the reference and the NIPTES-containing coatings revealed that less F but more C and O were detected after the introduction of NIPTES. This correlates well with the chemical structure of the fluorescent marker (see Figure 4), which comprises hydrocarbon units in the aromatic ring system and some oxygen in the imide groups. In accordance with the applied ratio of PFOS: BOS: NIPTES (see Table 1), all samples prepared from coat_1 showed lower F and higher N concentrations at their surface than the coat_2 samples. Additionally, the C, O and Si concentrations were slightly higher for coat_1 compared to coat_2.

When comparing the experimentally found compositions to the theoretical values (cf. Table 2), it becomes evident that the detected F content exceeds the calculated amount in all samples (reference, as well as coat_1 and coat_2 layers). This was explained by the well-known phenomenon of surface rearrangement (or segregation) behavior of low surface energy compounds (e.g., fluorinated components), which are, although covalently bound to a polymer network, preferably exposed to the free surface in a non-polar environment (e.g., air) to minimize the interfacial free energy of the system [57,58,59]. Concerning the organosilane coatings of this work, this means that the perfluorinated units of PFOS orientate toward the surface, which is reflected by the increased F concentrations. Moreover, the carbon contents obtained from XPS measurements were lower than the calculated values. This was interpreted as a further consequence of the preferred exposure of PFOS to the surface, since XPS is a surface-sensitive technique, and the majority of hydrocarbon units belong to BOS and the aromatic system of NIPTES. Moreover, the experimentally determined amounts of O and N are in good agreement with the calculated value, while for Si, a deviation from the expected contents was observed.

Within the series of coat_1 samples, increasing temperatures during evaporation of the solvent caused significantly decreased F (from 30 to 20 at%) but increased C (from 48 to 52 at%), O (from 13 to 15.5 at%), Si (from 8 to 9.5 at%) and N (from 1.5 to 2.4 at%) concentrations at the surface of the coatings. In contrast, the composition of coat_2 samples was rarely affected by changes in evaporation temperature. Again, this was attributed to surface rearrangements (segregation behavior) within the coating, leading to the preferred exposure of the perfluorinated moieties of PFOS to the free surface. This mechanism seems to work efficiently in coat_2 samples and the reference coatings, while the movement and the resulting exposure of the perfluorinated chains of PFOS in coat_1 appear to be hindered to a certain extent. As described in Ref [57], the mobility of the perfluorinated chains depends on the chain length of the fluorinated compound, as well as on the stiffness of the polymer matrix. Based on these findings, the reduced chain mobility in coat_1 was assigned to an increased rigidity of the siloxane network, as a consequence of the high amounts of NIPTES in this formulation (see Table 1), and the presence of a multitude of bulky naphthalimide groups. On the contrary, coat_2 and the reference contained less or no NIPTES, respectively, which resulted in a less restricted chain mobility.

### 3.3. Application-Releated Characteristics of the Coatings

**Figure 7:**
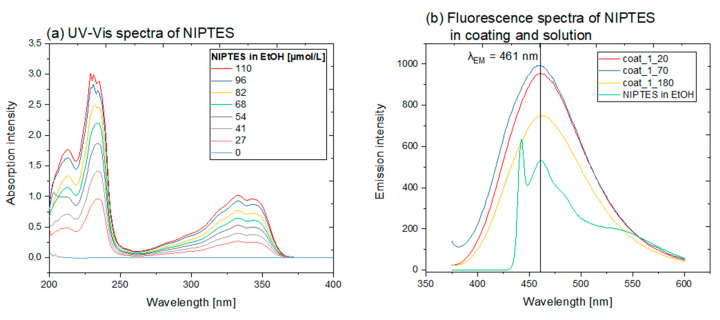
(**a**) UV-Vis absorption spectra of NIPTES dissolved in ethanol at various concentrations and (**b**) fluorescence spectra of coat_1 samples containing NIPTES in the organosilane coating and of NIPTES in ethanol solution (excitation wavelength λ = 365 nm).

As mentioned above, the naphthalimide moiety of NIPTES provided the required visibility-on-demand property (fluorescence) to the coatings. This was evidenced by UV-Vis spectroscopy of the synthesized NIPTES dissolved in ethanol, revealing a strong absorption by the marker molecule in the range of 220–240 nm as well as in the region from 310 to 370 nm (see Figure 7a). As a consequence of UV absorption, a blue fluorescent emission with its maximum at 461 nm was detected for both solutions of NIPTES in ethanol as well as NIPTES-containing coatings on silicon wafers (see Figure 7b). This fluorescence was detected for all the investigated organosilane layers containing NIPTES, independent of the variations in sample preparation (e.g., evaporation temperature). The different shapes of the fluorescence spectra of the NIPTES solution and the NIPTES-containing coatings were attributed to the different concentrations in solution and matrix, and to the influence of the solid matrix. Moreover, it has to be mentioned that a wavelength of 365 nm was chosen for excitation in the fluorescence measurements, although this does not match with the absorption maximum determined by UV-Vis analysis. This decision was based on the fact that light in the UV-A region was seen as most suitable for practical applications, since it has a lower potential to harm the eyes and skin and is available as a hand-held flashlight, avoiding bulky equipment. However, the observed absorption and emission characteristics suggest that NIPTES is suitable as a marker molecule in organosilane layers, providing blue fluorescence under UV irradiation.

**Figure 8:**
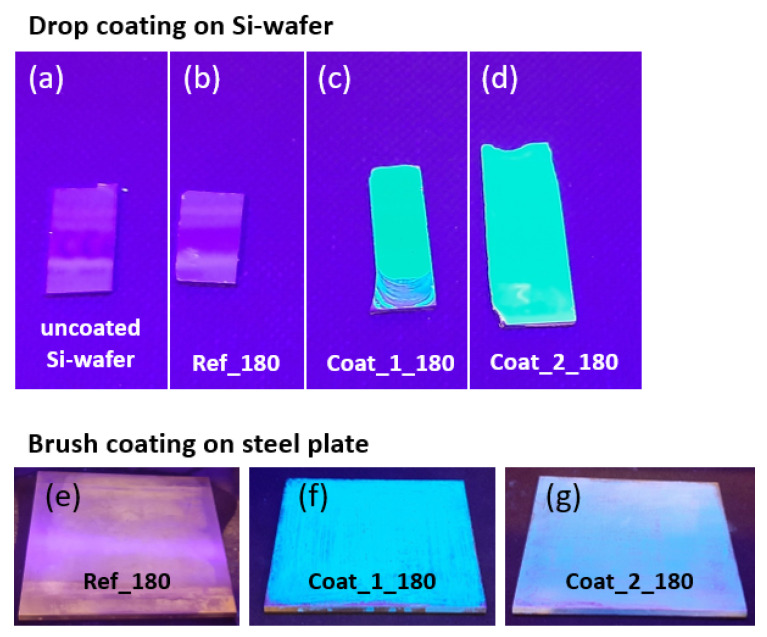
Images of uncoated and coated Si wafers and steel plates under UV irradiation (λ = 365 nm); top row: (**a**) uncoated Si wafer, (**b**) ref_180, (**c**) coat_1_180 and (**d**) coat_2_180 on Si wafers; bottom row: (**e**) ref_180, (**f**) coat_1_180 and (**g**) coat_2_180 on steel plates.

The emission in the blue region discussed above was also visible with the eye, as demonstrated in Figure 8, showing the uncoated and coated silicon wafers as well as the exchangeable steel plates for the injection mold. While the uncoated substrates and the reference coating (based on PFOS and BOS) did not fluoresce under UV irradiation with λ = 365 nm, the NIPTES-containing coatings exhibited a significant blue fluorescence all over the coated area, independent of the chemical composition of the coatings. This demonstrates the potential of NIPTES as a suitable fluorescent marker, enabling the required temporary quality control of organosilane-based coatings deposited on solid surfaces.

**Figure 9:**
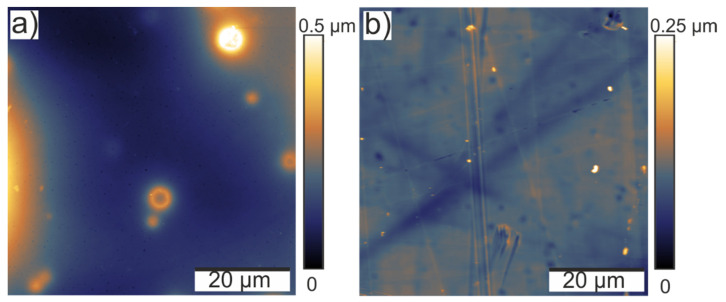
AFM images of (**a**) a drop-coated and (**b**) a brush-coated layer of coat_1_180 on a Si wafer and a steel plate, respectively.

The morphology of the neat substrates, as well as the applied coatings, was examined by means of AFM. The obtained root mean square values (RMS, see Table 3) provide information about the roughness of all the uncoated and coated substrates. A comparison of the drop-coated Si wafers and brush-coated steel plates revealed that the roughness of the deposited organosilane layers was significantly influenced by the application method. Although the RMS of the uncoated silicon wafers (1.8 nm) was lower than the RMS of the steel substrates (65 nm), the drop-coated layers showed distinctly higher roughness (RMS = 195–360 nm) and more inhomogeneities (represented by higher standard deviations) than their brush-coated counterparts (RMS 14 and 93 nm, respectively). This is also illustrated in Figure 9, as an example of drop- and brush-coated layers of coat_1_180.

Moreover, the impact of the chemical composition on the roughness of the coatings was evaluated. As can be seen in Table 3, the drop-coated reference coatings prepared at 20 and 180 °C were significantly rougher than the corresponding coat_1 and coat_2 samples, while the coatings prepared at 70 °C showed a lower RMS. In general, it was concluded that the introduction of NIPTES led to smoother coating surfaces, which, in turn, means that the roughness increased with increasing PFOS concentrations (ref > coat_2 > coat_1). This trend was even more pronounced for the brush-coated samples. In more detail, coat_1_180 seemed to fill up the structures of the steel plates, since the original RMS of 65 nm was reduced to 14 nm, while coat_2_180 and ref_180 exhibited higher RMS values (93 nm and 120 nm, respectively).

Concerning the impact of the evaporation temperature on the RMS, no trend was observed for the series of reference coatings. On the contrary, the coat_1 layers prepared at 20 and 70 °C showed higher RMS values (309 and 330 nm) than the respective coating deposited at 180 °C (195 nm). A similar trend was found for the series of coat_2, in which the roughness decreased with increasing evaporation temperatures (see Table 3).

In addition to morphology, the adhesion force of the applied coatings was also assessed by means of AFM. As the coating was much softer than the steel samples, the measured adhesion forces (pull-off forces) were normalized to the effective contact area in order to be comparable. The contact area *A* can be estimated according to the following equation:

(1) A=π·[34·F·R·(1−v12E1+1−v22E2)]23

Here, *F* is the contact force (tip load), *R* is the tip radius of the curvature, *E_i_* and *v_i_* are the Young moduli and Poisson ratios of the tip and the sample materials, respectively [60].

The tip load was 23.8 nN for all adhesion force measurements. The tip radius of the curvature was 7 nm, according to the specifications of the supplier. Young’s modulus and Poisson ratio of the silicon tip were assumed to be 160 GPa and 0.22 [61]; the values for steel were 200 GPa and 0.3 [62]. For the coating, no literature values were available. Therefore, Young’s modulus was estimated from AFM nanoindentation experiments. For the Poisson ratio of the coating, a value of 0.5 was assumed. The force versus distance curves were evaluated according to a Hertz contact model [63], yielding a Young modulus of (0.45 ± 0.02) GPa. The normalized adhesion forces are compiled in Table 4.

The obtained results prove the anti-adhesive effect of the organosilane coatings. The initial adhesion force of 2.35 nN/nm^2^—measured on the uncoated steel plates—was significantly reduced to 0.25 nN/nm^2^ by the application of the coating ref_180, and to 0.16 nN/nm^2^ and 0.19 nN/nm^2^ with coat_1_180 and coat_2_180, respectively. Since the adhesion forces of the fluorescent coatings are in the same order of magnitude as the adhesion force of the reference coating, it can be concluded that the anti-adhesive effect is still maintained after the incorporation of NIPTES. However, no correlation between the PFOS ratio in the coating formulations and the adhesion forces was observed, since the latter varied on too small a scale.

In addition, the contact angle measurements with water corroborated the findings of the adhesion force measurements. As can be derived from Table 5, the uncoated substrates (Si wafer and steel plates) exhibited water contact angles (WCA) of 60° (prior to activation). The application of the reference coating based on PFOS and BOS caused a significant increase in the WCA to 105° on Si wafers and 116° on steel plates, respectively. This is explained by the already established relation of the anti-adhesive effect and the immobilization of perfluorinated compounds at the substrate surface [10].

On the coated steel plates, a clear trend toward higher WCA was observed for an increasing fraction of fluorinated groups in the coatings (ref_180 > coat_2_180 > coat_1_180). This correlates well with the findings of the XPS analysis, where increased PFOS ratios led to increased fluorine concentrations on the coated surfaces (see Table 2). In contrast, the WCA for all coatings on the Si wafers varied between 104 and 106°, independent of the coating composition. The missing trend on Si wafers was explained by the impact of surface roughness on the contact angle [64,65], which was distinctly higher for drop-coated organosilane layers (Si wafers) than for the brush-coated layers (steel plates), as already discussed with the AFM results (see Table 3). Practically, this means that it was possible to display large differences in the WCA, as obtained for uncoated and coated wafers, while smaller changes, as obtained by variation of the coating compositions, could not be distinguished.

Based on the XPS results, which revealed considerably decreased F contents with increasing evaporation temperatures (see Table 2), the effect of the evaporation temperature on the WCA was investigated within the series of coat_1 on Si wafers. However, again, the high surface roughness of the applied coatings impeded a detailed evaluation within the required WCA range.

Finally, the control function based on the visibility-on-demand property of the fluorescent coatings was demonstrated along an injection molding process. Here, plate-shaped polymer parts were produced from a blue chalk-filled polypropylene compound. The combination of a chalk-filled polymer and the harsh processing conditions, including the high injection pressure (550 bar) and particularly the high pressure acting on the produced part during demolding (44 bar), posed a particular challenge to the coatings, thus provoking (the intended) fast abrasion and wear of the organosilane coatings on steel (1.5–2.5 µm).

**Figure 10:**
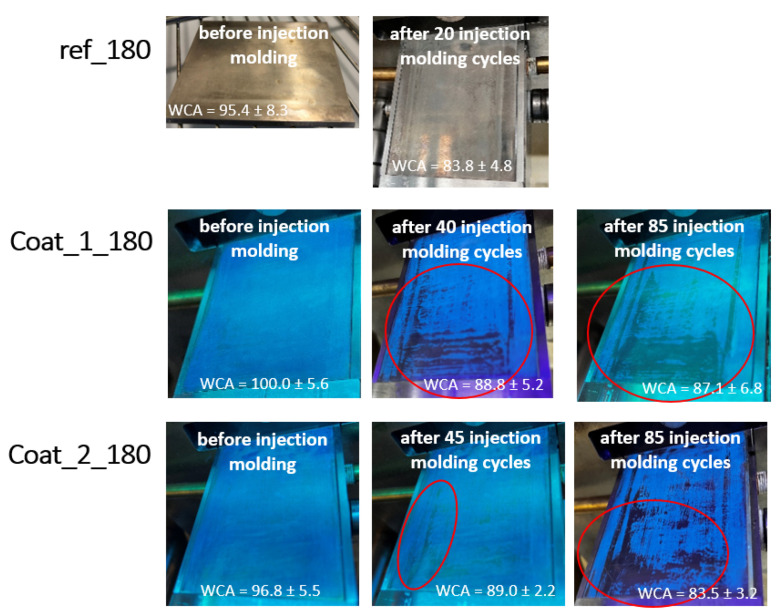
Demonstration of abrasion and wear of the coatings ref_180, coat_1_180 and coat_2_180 on steel plates during the injection molding process. The corresponding water contact angles (WCA) are given at the bottom of each image.

The mechanical degradation of ref_180, coat_1_180 and coat_2_180 was followed by irradiation with a UV lamp and contact angle measurements with water after every 10th production cycle (see Figure 10). It is clearly visible that the incorporated fluorescent marker NIPTES provided the required quality control on demand, while the damage of the reference coat (ref_180) could not be monitored. Increasing abrasion and wear of coat_1_180 and coat_2_180 with increasing number of injection molding cycles were observed, mainly in the region where the highest pressure was applied during demolding (first 30 mm of the substrate). Moreover, the worn lines occurring at the edges of the substrate were caused by closing of the cavity, where the vertical piston contour directly contacts the coated steel substrate. A comparison of the fluorescent coatings after 40–45 and after 85 injection molding cycles showed that coat_1_180 was more rapidly abraded than coat_2_180, despite its lower roughness (see Table 3). A possible explanation was the lower layer thickness of coat_1_180, which was estimated to be approx. 1 µm lower in thickness than coat_2_180. In addition, coat_2_180 appeared to be more scratch resistant during the profilometry experiments.

The mechanical degradation of the coatings was accompanied by a decrease in WCA, as noted in Figure 10. The initial WCA values of 95–100° were reduced to 83–87° after only 20 injection molding cycles for ref_180, but it took 85 cycles for coat_1_180 and coat_2_180 to achieve the same effect. These results suggest that the introduction of NIPTES improved the abrasion stability of the original anti-adhesive coating based on PFOS and BOS (reference). Concerning the comparatively low number of production cycles, which the coatings resisted, the authors would like to point out the combination of a chalk-filled polymer with the very harsh conditions during this injection molding process. Moreover, it is worth mentioning that the starting WCA (before injection molding) shown in Figure 10 do not coincide with the values listed in Table 5. This is explained by the well-known influence of temperature on the contact angle of a liquid [66] and the fact that the substrates were tempered at 30 °C for the injection molding experiments.

## 4. Discussion

The incorporation of the fluorescent marker 1,8-naphthalimide-N-propyltriethoxysilane (NIPTES) into an anti-adhesive organosilane coating was achieved via its ethoxy groups. This was proven both by FTIR spectroscopy as well as XPS analyses, which revealed a nitrogen content of 1.5–2.5 at% and signals at 288 eV and 400 eV in the C1s and N1s spectra, as well as typical imide bands at 1710–1590 cm^−1^ (C=O of imides) for the coatings containing NIPTES. The characterization of the coatings with UV-Vis and fluorescence spectroscopy evidenced the absorbance by the naphthalimide unit of NIPTES in the UV range (220–240, 310–360 nm) and fluorescent emission in the blue region (λ_max_ = 463 nm). AFM measurements revealed that the incorporation of NIPTES improved the homogeneity of the coatings (decreased RMS) and also showed that the anti-adhesive property of the original formulation (adhesion force = 0.25 nN/nm^2^ versus 2.35 nN/nm^2^ for uncoated steel) was still maintained after the incorporation of the fluorescent marker (adhesion force = 0.16–0.19 nN/nm^2^). These findings were supported by the high WCA for all coated substrates, ranging from 107 to 116 °, increasing with the fraction of PFOS in the formulation. Finally, the introduced control function was demonstrated practically, visualizing the coatings’ qualities along an injection molding process utilizing the fluorescence of NIPTES. Moreover, these experiments indicated an improved abrasion stability for NIPTES-containing coatings.

To sum up, the required visibility-on-demand property was introduced successfully without impairing the primary character of the organosilane layer, especially its anti-adhesive property and low surface energy. In addition, the incorporation of NIPTES improved the homogeneity and abrasion stability of the original coating.

Based on these findings, the developed fluorescent anti-adhesive coating entails several advantages for practical and industrial applications:

- The visibility-on-demand property provides a straightforward, quick and temporary quality control of coated surfaces by using a UV lamp.
- Moreover, this approach provides a facile and smart way to access information about the presence and the homogeneity, as well as about the abrasion and wear of the coating at any time during application and at any stage of the industrial processes.
- While conventional methods, such as microscopy, spectroscopy, interferometry, etc., often rely on the characterization of representative spots on the sample, the introduction of NIPTES as a fluorescent marker allows for the inspection of the entire coated area.
- The convenient use of a hand-held UV lamp renders it possible to easily implement the developed concept for an on-demand quality control in industrial processes, without the use of bulky equipment and complex evaluation procedures.
- The organosilane coating is applied in a simple and rapid procedure. The incorporation of NIPTES does not require any additional step or changes to the approved deposition procedure, since the fluorescent marker is simply added to the formulation and deposited together with the organosilanes of the basic coating.

Due to the combination of the visibility-on-demand property and the simple and rapid application procedure, the authors see great potential for the developed anti-adhesive organosilane coating in industrial applications, in which it is important to rely on long service intervals and to provide an economic workflow (e.g., as a permanent demolding aid in the production of polymers).

## Figures and Tables

**Table 1:** Chemical composition of and preparation conditions for all prepared samples.

Sample Name	Molar Ratio of PFOS: BOS: NIPTES	Time for Prehydrolysis [h]	Temperature for Solvent Evaporation [°C]
**ref_20**	4.4: 1: 0	48	20
**ref_70**	4.4: 1: 0	48	70
**ref_180**	4.4: 1: 0	48	180
**coat_1_20**	1.5: 1: 7	24	20
**coat_1_70**	1.5: 1: 7	24	70
**coat_1_180**	1.5: 1: 7	24	180
**coat_2_20**	4.4: 1: 2.4	48	20
**coat_2_70**	4.4: 1: 2.4	48	70
**coat_2_180**	4.4: 1: 2.4	48	180

**Table 2:** Chemical composition of reference coatings and coatings containing NIPTES (all data refer to the surface composition in atom-% calculated from XPS data; hydrogen is omitted).

	Chemical Composition [at%]				
	F	C	O	Si	N
**ref_20**	46.2 ± 1.9	37.2 ± 0. 0	9.4 ± 0.8	7.3 ± 1.1	/
**ref_70**	46.8 ± 0.6	37.0 ± 0.3	8.8 ± 0.2	7.4 ± 0.2	/
**ref_180**	47.1 ± 0.2	33.4 ± 2.8	10.9 ± 0.7	8.7 ± 2.3	/
**ref (theor.)**	34.8	49.6	11.7	3.9	/
**coat_1_20**	30.1 ± 0.8	48.0 ± 0.9	12.8 ± 0.3	7.7 ± 0.6	1.5 ± 0.2
**coat_1_70**	23.9 ± 0.5	51.0 ± 0.2	14.3 ± 0.3	8.6 ± 0.2	2.2 ± 0.2
**coat_1_180**	20.6 ± 0.7	51.9 ± 2.8	15.6 ± 1.6	9.5 ± 2.3	2.4 ± 0.5
**coat_1 (theor.)**	6.9	69.7	16.9	3.9	2.6
**coat_2_20**	32.2 ± 0.1	48.0 ± 0.2	11.5 ±0.2	6.7 ± 0.3	1.5 ± 0.2
**coat_2_70**	32.1 ± 1.6	48.5 ± 1.1	11.6 ± 0.4	6.0 ± 0.2	1.7 ± 0.3
**coat_2_180**	31.8 ± 1.9	47.4 ± 1.5	12.1 ± 0.2	7.0 ± 0.3	1.7 ± 0.2
**coat_2 (theor.)**	24.8	56.9	13.5	3.8	1.0

**Table 3:** Root mean square values of Si wafers and steel plates prior to activation and coating, as well as coated with the reference coating, coat_1_and coat_2. The samples were prepared at evaporation temperatures of 20, 70 and 180 °C, respectively.

Sample	RMS [nm]
**uncoated Si wafer**	1.8 ± 0.4
**ref_20 on Si wafer**	539 ± 90
**ref_70 on Si wafer**	250 ± 26
**ref_180 on Si wafer**	1433 ± 475
**coat_1_20 on Si wafer**	309 ± 175
**coat_1_70 on Si wafer**	330 ± 218
**coat_1_180 on Si wafer**	195 ± 40
**coat_2_20 on Si wafer**	360 ± 175
**coat_2_70 on Si wafer**	290 ± 153
**coat_2_180 on Si wafer**	197 ± 143
**uncoated steel plate**	65 ± 26
**ref_180 on steel plate**	120 ± 10
**coat_1_180 on steel plate**	14 ± 30
**coat_2_180 on steel plate**	93 ± 85

**Table 4:** Adhesion forces obtained from the AFM pull-off force measurements (normalized to the estimated contact area).

Sample	Normalized Adhesion Force [nN/nm^2^]
**uncoated steel plate**	2.35 ± 0.77
**ref_coating_180**	0.25 ± 0.08
**coat_1_180**	0.16 ± 0.02
**coat_2_180**	0.19 ± 0.02

**Table 5:** Water contact angles of Si wafers and steel plates prior to activation and coating as well as coated with ref_180, coat_1_20, coat_1_70, coat_1_180 and coat_2_180.

Sample	Water Contact Angle [°]
**uncoated Si wafer**	60.6 ± 3.9
**ref_180 on Si wafer**	105.5 ± 3.7
**coat_1_20 on Si wafer**	105.3 ± 2.7
**coat_1_70 on Si wafer**	103.2 ± 2.0
**coat_1_180 on Si wafer**	104.5 ± 5.4
**coat_2_180 on Si wafer**	106.2 ± 0.4
**uncoated steel plate**	60.3 ± 3.9
**ref_180 on steel plate**	116.1 ± 2.8
**coat_1_180 on steel plate**	107.1 ± 0.9
**coat_2_180 on steel plate**	112.4 ± 1.4

## Data Availability

Not applicable.
